# The Hidden Cost of Precision: Financial, Training, and Environmental Trade-Offs in Robotic HPB Surgery

**DOI:** 10.1007/s11701-026-03404-7

**Published:** 2026-04-21

**Authors:** Basil Habash

**Affiliations:** University of Debrecen, Debrecen, Hungary

**Keywords:** Robotic surgery, Hepatopancreatobiliary surgery, Economic considerations, Learning curve, Environmental impact, Narrative review

## Abstract

Robotic surgery has redefined hepatopancreatobiliary (HPB) practice, delivering enhanced precision and promising improved patient outcomes. Yet these benefits come with hidden costs, which are crucial in shaping adoption, equitable access, and hospital workforce capacity. This narrative review examines these hidden costs across three domains: financial, training, and environmental. From a financial perspective, we assess procedural cost structures, capital investment, market dynamics, reimbursement, and opportunity costs. In terms of training, we explore learning curves, their consequences, and the structural training limitations hindering skill diffusion. On the environmental front, we evaluate the ecological impact of robotic surgery and the importance of comprehensive life-cycle analyses. In addition, the review highlights areas that warrant further research and explores strategies to address the identified hidden costs. Collectively, the findings underscore the need to evaluate robotic HPB surgery not only for clinical outcomes, but also for its broader system-level implications to ensure equitable, sustainable, and economically prudent adoption.

## Introduction

Hepatopancreatobiliary (HPB) surgery encompasses some of the most technically complex abdominal procedures, owing to the intricate vascular and biliary anatomy of the liver and pancreas, diverse pathology, and substantial morbidity rates. In the 1990s, minimally invasive techniques entered the HPB arena, and laparoscopic approaches rapidly spread world-wide on the promise of reduced blood loss, shorter hospital stays, and faster recovery [1]. However, despite offering clear benefits to patients, laparoscopy increased the technical burden on HPB surgeons, who were challenged by two-dimensional visualization, awkward ergonomics, rigid instruments, and fulcrum effects [2].

At the turn of the millennium, robotic surgery platforms were introduced to overcome these limitations, offering three‑dimensional stereoscopic vision, wristed instruments with multiple degrees of freedom, enhanced ergonomics, and tremor filtration [3]. The clinical impact of these technical advances is becoming increasingly apparent: high-level evidence suggests that robotic HPB surgery is associated with more favorable perioperative outcomes than laparoscopy across multiple endpoints, including textbook outcomes, conversion rates, blood loss, morbidity, and oncologic margins [4–8].

Accordingly, the adoption of robotic HPB surgery has increased substantially in recent years. For example, in a nationwide study from the Netherlands, the proportion of liver surgeries performed robotically increased from 0.2% in 2014 to 11.9% in 2020 [9]. Similarly, in the United States, the proportion of distal pancreatectomy done robotically for pancreatic adenocarcinoma rose from 2.2% in 2010 to 19.4% in 2019 [10].

In light of growing clinical enthusiasm, published literature has largely focused on feasibility, perioperative outcomes, and short-term comparative metrics. The broader implications of adopting robotic HPB surgery remain underexplored, with key questions around financial sustainability, training requirements, and environmental impact left largely unanswered. Such considerations are increasingly relevant as healthcare systems face fiscal constraints, workforce pressures, and rising expectations for environmental responsibility.

This narrative review synthesizes current evidence on the hidden financial, training, and environmental trade-offs accompanying the adoption of robotic HPB surgery. Limitations within the existing literature are highlighted, and potential strategies to mitigate identified challenges are discussed, with the aim of informing balanced, sustainable, and equitable integration of robotic technology into HPB practice.

## Methods

This narrative review was conducted to provide a structured overview of the financial, training, and environmental implications of robotic HPB surgery.

A literature search was performed using PubMed to identify relevant articles published up to February 2026. The search combined keywords related to robotic HPB surgery and the three thematic domains of the review. Core terms included “robotic surgery,” “hepatopancreatobiliary” (or “HPB”), and organ- and procedure-specific terms such as “liver,” “pancreas,” “resection,” “hepatectomy,” and “pancreatectomy.” Additional terms reflecting the thematic focus included “cost,” “economic evaluation,” “training,” “learning curve,” “environmental impact,” and “carbon footprint.” Truncated terms (e.g., “hepat*” and “pancreat*”) were used where appropriate to capture variations in terminology. Reference lists of selected articles were also manually screened to identify additional relevant studies.

Studies were selected based on their relevance to the thematic focus of the review. Both original research articles and review papers were included to provide a comprehensive perspective.

### Financial implications

Decisions surrounding the adoption of robotic HPB surgery are shaped by financial considerations at the procedural, institutional, and health-system levels. Studies have consistently demonstrated higher intraoperative costs associated with robotic HPB surgery than with laparoscopic and open approaches across a broad range of procedures, including hepatectomy [11–13], pancreatic resections (distal pancreatectomy and pancreaticoduodenectomy) [14–18], and subtotal cholecystectomy [19]. These cost differentials are mostly attributable to longer operative times, especially earlier in the learning curve, and disposable instruments and accessories [20].

However, the picture is less consistent when considering the overall cost of robotic HPB procedures compared with laparoscopic and open approaches. In pancreatic resections, Lyu et al. showed that intraoperative expenditure represents only a relatively small proportion of total hospital cost [16]. Once factors such as length of hospital stay, postoperative complications, intensive care unit (ICU) utilization, long-term recurrence, and patient quality-adjusted life years (QALYs) are included, financial outcomes across studies are heterogeneous (Table 1): some series report lower total costs for robotic procedures [12, 13, 16, 17, 21], others find comparable expenditures [15, 19, 22], and still others observe higher total costs [11, 12, 14, 18, 23, 24]. The matter is further complicated by the methodological challenges of capturing costs beyond operative and hospitalization expenses, including ancillary staff, rehabilitation, and productivity loss following surgery [25]. Despite the aforementioned heterogeneity at the level of individual studies, available meta-analyses suggest a more consistent direction of effect. Robotic liver surgery and distal pancreatectomy procedures were shown to entail higher overall costs than conventional approaches, with per-procedure cost premiums on the order of several thousand USD [8, 26, 27].

A key limitation explicitly noted in prior cost analyses is the exclusion of capital acquisition, amortization, and service costs of robotic platforms [14, 21]. This omission is understandable, as allocation of capital costs is challenging when a single robotic platform serves multiple specialties [28]. Nevertheless, these expenditures can be substantial and should not be ignored. For example, Childers et al. analyzed the financial statements of Intuitive Surgical Inc., the primary supplier of robotic surgical systems, and estimated that in 2017 per-procedure costs of robotic platforms averaged USD 1,038 for acquisition and USD 663 for service contracts [29]. Although averaged across specialties and centers, these figures underscore the considerable financial burden of robotic systems. Importantly, capital costs are fixed and are borne irrespective of case volume, which may disproportionately impact lower-volume HPB centers. Furthermore, these costs are heavily influenced by market dynamics, where, according to market analysis reports, Intuitive Surgical Inc. currently maintains a near-monopolistic position, effectively shaping pricing and access for hospitals [30]. The emergence of additional robotic system manufacturers in the coming years may reduce per-procedure capital costs, increase hospital purchasing power, and foster innovation in the field [31, 32].

At the institutional level, a further challenge is that hospital-borne costs for robotic surgery can be misaligned with reimbursement from public and private payers. Reimbursement analyses across surgical specialties suggest that robotic procedures are typically reimbursed at rates comparable to conventional approaches, despite higher technology-related costs incurred by hospitals [33]. This mismatch thereby shifts financial risk to institutions and may limit incentives for adopting robotic surgery.

At a system level, opportunity cost is a critical consideration in the economics of robotic HPB surgery. Resources allocated to robotic platforms, such as capital investment, operating room capacity, and recurrent purchase of instruments with restricted reuse cycles, are inherently unavailable for alternative and potentially more clinically impactful areas such as enhanced recovery after surgery (ERAS) programs or workforce development. For illustration, a meta-analysis of procedures from multiple surgical specialties found that the increased operative time of robotic surgery alone represents an average opportunity cost of approximately €490 per case [34]. Nonetheless, it is important to recognize that opportunity cost is bidirectional. In practice, shorter hospital stays after robotic procedures may reduce opportunity losses associated with occupied beds that can be reassigned to patients with greater clinical urgency or complexity [20]. This reframes robotic HPB surgery not simply as a higher-cost intervention, but as a strategic allocation decision within finite health system resources.

**Table 1:** Comparative cost outcomes in robotic, laparoscopic, and open HPB surgery

Study	Procedure	Comparison	Overall Cost	Drivers of Cost Difference
Miller et al. [11] (*n* = 454)	Hepatectomy	Robotic vs. Laparoscopic	Higher for robotic	Increased blood transfusions, length of stay, and OR time
Xia et al. [12] (*n* = 2142)	Hepatectomy for BCLC 0/A hepatocellular carcinoma	Robotic vs. Laparoscopic	Higher for robotic	Higher intraoperative cost
		Robotic vs. Open	Lower for robotic	Lower long-term treatment costs outweigh higher intraoperative cost
Hawksworth et al. [13] (*n* = 200)	Hepatectomy	Robotic vs. Open	Lower for robotic	Reduced length of stay and ICU utilisation outweigh higher OR cost
De Pastena et al. [14] (*n* = 103)	Distal Pancreatectomy	Robotic vs. Laparoscopic	Higher for robotic	Higher intraoperative cost
Wakabayashi et al. [15] (*n* = 120)	Pancreatoduodenectomy	Robotic vs. Open	Comparable	Higher intraoperative cost offset by reduced postoperative complications and length of stay
Lyu et al. [16] (*n* = 264)	Pancreatectomy for any diagnosis	Robotic vs. Open	Lower for robotic	Reduced postoperative inpatient costs and length of stay outweigh higher OR cost
Rodriguez et al. [17] (*n* = 89)	Distal Pancreatectomy	Robotic vs. Laparoscopic	Higher for robotic	Higher OR materials and occupation costs
		Robotic vs. Open	Higher for robotic	Higher OR materials and occupation costs
Boggi et al. [18] (*n* = 238)	Pancreatoduodenectomy	Robotic vs. Open	Higher for robotic	NA
Caldwell et al. [19] (*n* = 126)	Subtotal fenestrating cholecystectomy	Robotic vs. Laparoscopic	Comparable	Higher intraoperative cost offset by reduced postoperative care cost
D’Hondt et al. [21] (*n* = 311)	Liver surgery (resections)	Robotic vs. Laparoscopic	Lower for robotic	Reduced instruments and accessory cost
Vicente et al. [22] (*n* = 59)	Distal Pancreatectomy	Robotic vs. Laparoscopic	Comparable	No significant difference in cost, but higher QALYs for robotic procedure
Xu et al. [23] (*n* = 42)	Radical resection for hilar cholangiocarcinoma	Robotic vs. Open	Higher for robotic	NA
Butturini et al. [24] (*n* = 43)	Distal Pancreatectomy	Robotic vs. Laparoscopic	Higher in robotic	Higher intraoperative cost

n: total number of patients included in the study (after propensity score matching where applicable)

OR, Operating Room; ICU, Intensive Care Unit; NA, Not Available/Reported; QALY, Quality-Adjusted Life Year

## Training implications

Attaining proficiency in robotic HPB surgery is no simple task; it entails a learning process that demands time, case exposure, mentorship, and institutional resources. In the literature, a learning curve is typically defined as the number of cases required for key performance metrics to improve and reach a stable plateau, with operative time, blood loss, conversion, and postoperative complications most commonly evaluated [35]. Reports of learning curves vary widely by procedure type, case complexity, and the metrics used to define the learning curve (Table 2). For example, pooled data indicate that initial proficiency can be achieved after 20–30 cases for robotic distal pancreatectomy, and after 40–60 cases for robotic pancreatoduodenectomy [36]. For robotic liver resections, a high-volume single-surgeon series shows that proficiency was achieved after 20 cases, whereas progression to mastery required a markedly greater experience of 65 cases [37]. Other series report still higher mastery thresholds, reaching 100 cases for major resections [38].

The absence of a gold standard for evaluating learning curves complicates the interpretation of findings across studies. Nonetheless, the scale of learning curves in robotic HPB surgery carries important system-level and ethical implications. For instance, low-volume centers may be unable to accrue sufficient case volumes to complete learning curves of this magnitude, potentially leading to abandonment of the robotic program and referral of cases to higher-volume centers. This dynamic can accelerate centralization of care, thereby limiting local access to robotic HPB surgery and exacerbating geographic and socioeconomic disparities in care [39]. Furthermore, ethical considerations arise during the early phases of adoption. Multiple studies have demonstrated transiently increased postoperative morbidity during the initial stages of the learning curve [38, 40–43], raising concerns regarding patient exposure to elevated procedural risk despite the availability of established, lower-risk alternatives such as laparoscopy. Importantly, emerging evidence suggests that these risks can be mitigated through mentorship and structured training programs, which have been shown to shorten learning curves and reduce complication rates [36, 44].

Notably, a widespread limitation exists across learning curve analyses: learning curves are reported solely as cumulative case numbers, without specifying case density or temporal spacing between procedures. Experimental evidence from robotic simulation illustrates that technical skill acquisition, retention, and decay are time-dependent, with longer gaps between practice associated with greater learning decay [45]. Clinically, a multicenter study of robotic liver resections found that surgeons who operated at least once a week achieved shorter learning curves and lower complication rates than those with lower procedure frequency [46]. Taken together, these findings suggest that simple case counts can mask the effects of procedural frequency and spacing, which may help explain variability across learning curve studies. The practical relevance of these dynamics is underscored by credentialing policies, whereby roughly one in four robotic surgery programs in the United States explicitly define maximum allowable gaps between procedures to maintain robotic privileges [47].

Beyond how quickly proficiency is achieved, the distribution of operative skill within robotic programs is also structurally constrained. Previous studies indicate that resident participation at the robotic console remains limited [48–50]. In qualitative interviews, general surgery residents reported that progression toward operative autonomy is hindered by barriers that are amplified or unique to robotic surgery education, most notably low case volume, infrequent exposure to robotic procedures, hierarchical training cultures, and attending comfort with both robotic surgery and console-based teaching [51]. Quantitative analyses further suggest that access to the robotic platform may be unevenly distributed, with disparities associated with resident gender and fellowship alignment [52]. Limited hands-on console experience may, in turn, slow the diffusion of technical skills to junior trainees, resulting in the concentration of advanced robotic expertise among fewer surgeons. Such concentration may have implications for workforce sustainability, including increased vulnerability to burnout, absence, and attrition.

Encouragingly, the very generation facing these training constraints may also be uniquely suited to robotic surgery, having grown up with early and intuitive exposure to digital technologies. Emerging evidence suggests that younger trainees demonstrate greater precision on robotic simulation tasks than older generations, with everyday digital behaviors as simple as two-thumb smartphone typing potentially conferring an advantage in early robotic skill acquisition [53]. Additionally, surgical education studies highlight dual-console systems as a potential solution, as they allow shared console access and graded transfer of operative control from attending to trainee [54, 55]. Taken together, these factors may support earlier resident autonomy and improved skill diffusion, helping to better prepare trainees for the demands of complex HPB practice.

**Table 2:** Summary of learning curves for robotic HPB procedures

Study	Procedure	Learning Stage*	Cases Required	Outcome Measures
Rahimli et al. [37]	Liver Resections	Proficiency	20	Operative time, case difficulty
		Early Mastery	30	
		Full Mastery	65	
Dugan et al. [38]	Major Liver Resection	Long-term Mastery	100	Operative time
	Minor Liver Resection		75	
	Technically Challenging Minor Liver Resection		57	
Jones et al. [40]	Pancreatoduodenectomy	Feasibility	30–45	Operative time, blood loss
		Proficiency	90	Postoperative pancreatic fistula grade B/C and major morbidity
Christodoulou et al. [41]	Pancreatoduodenectomy	Proficiency	88	Operative time
Ahmad et al. [43]	Major Hepatectomy	Proficiency	22	Operative time, blood loss
	Minor Heptectomy		34	

*Learning stages are listed as reported in the respective studies, and may not be directly comparable across studies given the absence of a standardized definition

## Environmental Implications

Healthcare systems must strike a balance between their professional obligation to optimize patient care, and their societal obligation to minimize environmental harm. Considerable evidence suggests that the intraoperative carbon and material footprint of robotic procedures surpasses that of conventional laparoscopic and open approaches across multiple surgical disciplines [56–58]. While data specific to HPB procedures are limited, evidence from gynecology, urology, and upper and lower gastrointestinal surgery provides important contextual insights. A recent systematic review surveying several surgical fields reported that robotic procedures produce up to 43.5% more greenhouse gas emissions, and up to 24% more solid waste compared with traditional laparoscopic alternatives [59]. Although extrapolation to HPB surgery must be undertaken cautiously, these findings may extend to selected robotic HPB procedures with operative complexity and duration comparable to those reported in the included studies.

The substantial energy demands of robotic platforms play a critical role in the environmental impact of robotic surgery. Comparative analyses have shown that robotic approaches consume significantly more electrical energy per procedure than both laparoscopic and open surgery. For example, Woods et al. demonstrated that robotic endometrial cancer staging required 46% more energy than laparoscopy and 81% more than laparotomy [60]. Of the total energy consumed per robotic procedure, 41% was attributed to the robotic platform itself [60]. These findings are echoed by Pastore et al., who reported that the da Vinci Xi system has an approximately 6-fold higher power demand than a standard laparoscopic tower, underscoring the intrinsic energy intensity of current robotic systems [61].

However, focusing exclusively on intraoperative emissions, such as those from instrument disposal and energy consumption in the operating room, provides an incomplete assessment of the true environmental impact of surgical care. A critical limitation of many surgical life cycle assessments is their failure to incorporate downstream emissions associated with postoperative recovery, particularly length of hospital stay. Addressing this gap, Pastore et al. evaluated total carbon emissions from robotic and laparoscopic radical prostatectomy, incorporating both procedural and postoperative phases [61]. Despite higher intraoperative energy consumption, the robotic approach was associated with lower overall CO₂ emissions, partly attributable to a significantly shorter postoperative hospital stay [61]. In that analysis, emissions related to postoperative inpatient care accounted for approximately one third of the total procedural carbon footprint [61]. Comparable data in HPB surgery remain sparse; however, robust evidence demonstrates that minimally invasive (robotic and laparoscopic) HPB surgery is associated with shorter postoperative hospital stays than open surgery [62, 63]. Within minimally invasive approaches, several comparative studies further suggest that robotic surgery may be associated with shorter hospital stays than conventional laparoscopy in selected HPB procedures, including distal pancreatectomy [64], right and extended right hepatectomy [65], and pancreatoduodenectomy [66, 67]. Considering that a single hospital bed-day is estimated to generate approximately 45 kg of CO₂-equivalent emissions and 5.5 kg of solid waste [68], reductions in postoperative length of stay may represent a pivotal determinant of the net environmental impact of robotic HPB surgery. Whether these downstream benefits are sufficient to offset the higher intraoperative emissions of robotic platforms remains uncertain and warrants procedure-specific investigation.

Beyond perioperative factors, opportunities exist to mitigate the environmental impact of robotic HPB surgery through targeted changes in surgical practice. One of the most effective strategies is the replacement of single-use disposable instruments with reusable or hybrid alternatives. Life cycle assessments consistently demonstrate that transitioning from single-use to reusable surgical equipment can reduce carbon emissions by more than 50% [69]. Specifically, hybrid robotic ports, which are predominantly reusable with limited disposable components, have been shown to possess an 83% lower carbon footprint than fully disposable ports [70]. Importantly, available evidence suggests that such transitions can be achieved without an increased risk of infection when appropriate sterilization protocols are followed, while potentially reducing overall procedural costs [71]. These findings highlight that, although robotic surgery is currently associated with a higher environmental burden, meaningful reductions are achievable through system-level and device-level interventions.

**Fig. 1:**
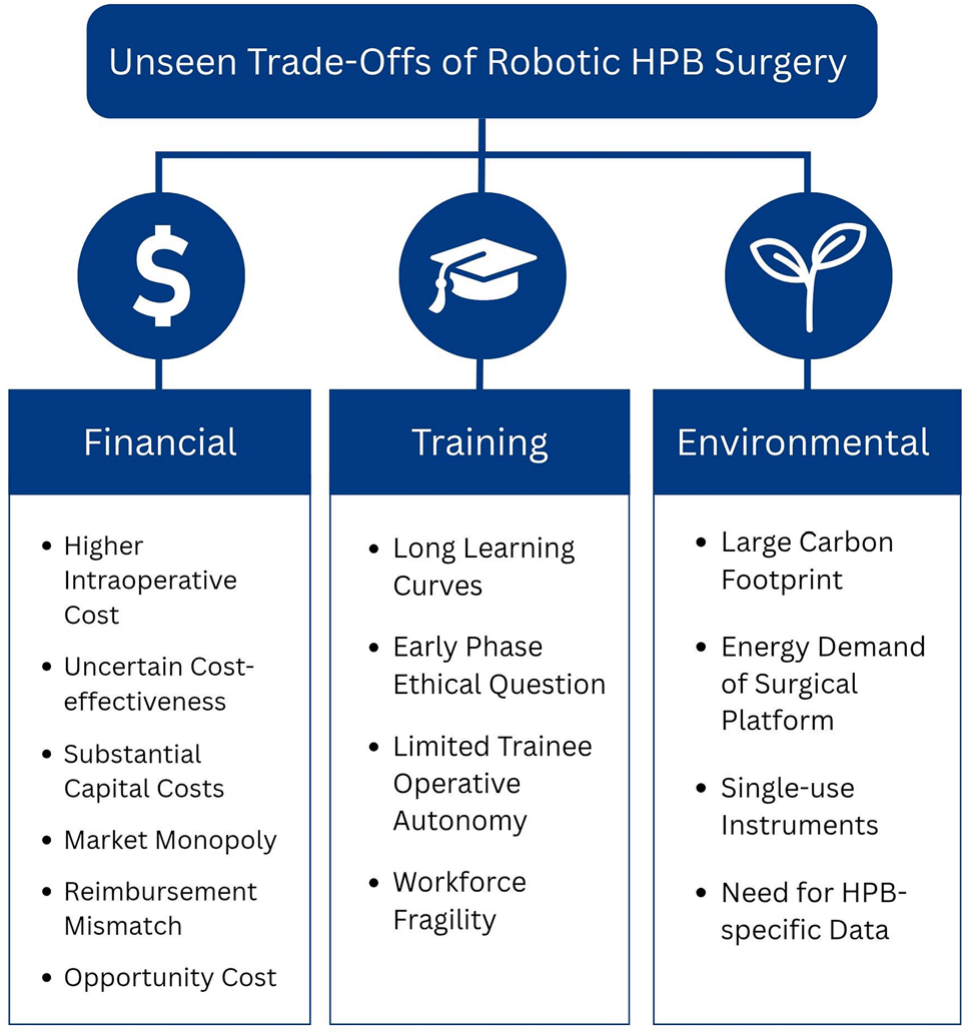
Summary of the hidden trade-offs surrounding the adoption of robotic HPB surgery

## Conclusion

Robotic surgery represents a meaningful technical advance in HPB practice. Although accumulating evidence supports favorable perioperative outcomes, these gains carry broader financial, training, and environmental trade-offs that influence accessibility, workforce capacity, and incentives for adoption (Fig. 1). These system-level ramifications extend beyond hospitals to affect patients, healthcare stakeholders, and society at large, so they must be weighed carefully.

The financial investment of adopting robotic surgery must fundamentally be weighed against the value of health – an inherently variable measure shaped by culture, resource availability, and societal priorities – while also balancing institutional budgets and stakeholder incentives. Likewise, the challenges of training and environmental impact raise ethical questions: how can innovation be advanced without imposing undue risk on patients or the planet? This dilemma is not new to advancing technology in healthcare, but it requires new solution pathways that specifically target the unique demands of robotic HPB surgery. Furthermore, such financial, training, and environmental burdens are particularly pronounced for smaller centers, where the operational scale may not be sufficient to absorb them. Centralization of robotic HPB surgery may therefore be inevitable; and while it concentrates expertise and accelerates innovation, it can also restrict access, highlighting the persistent tension between technology development and equitable care.

Still, these trade-offs remain insufficiently defined in many areas, as current literature is heterogeneous and frequently underreports capital investment, learning curve timelines, and comprehensive life-cycle environmental assessments. A better understanding of these overlooked factors can clarify the key challenges and guide the development of strategies to address them. Hence, future research should prioritize standardized cost analyses, structured training frameworks, and procedure-specific environmental assessments to ensure equitable, fiscally responsible, and environmentally conscious surgical advancement. The future of robotic HPB surgery must be defined not only by what technology enables, but by what healthcare systems can responsibly sustain.

## Data Availability

No datasets were generated or analysed during the current study.
